# Recent Advances in the Catalytic Conversion of Biomass to Furfural in Deep Eutectic Solvents

**DOI:** 10.3389/fchem.2022.911674

**Published:** 2022-05-09

**Authors:** Xu Zhang, Peng Zhu, Qinfang Li, Haian Xia

**Affiliations:** ^1^ Jiangsu Co-Innovation Center of Efficient Processing and Utilization of Forest Resources, College of Chemical Engineering, Nanjing Forestry University, Nanjing, China; ^2^ Jiangsu Provincial Key Lab for the Chemistry and Utilization of Agro-forest Biomass, College of Chemical Engineering, Nanjing Forestry University, Nanjing, China

**Keywords:** biomass, deep eutectic solvents, furfural, catalysis, solid acid

## Abstract

Lignocellulose is recognized as an ideal raw material for biorefinery as it may be converted into biofuels and value-added products through a series of chemical routes. Furfural, a bio-based platform chemical generated from lignocellulosic biomass, has been identified as a very versatile alternative to fossil fuels. Deep eutectic solvents (DES) are new “green” solvents, which have been employed as green and cheap alternatives to traditional organic solvents and ionic liquids (ILs), with the advantages of low cost, low toxicity, and biodegradability, and also have been proven to be effective media for the synthesis of biomass-derived chemicals. This review summarizes the recent advances in the conversion of carbohydrates to furfural in DES solvent systems, which mainly focus on the effect of adding different catalysts to the DES system, including metal halides, water, solid acid catalyst, and certain oxides, on the production of furfural. Moreover, the challenges and perspectives of DES-assisted furfural synthesis in biorefinery systems are also discussed in this review.

## 1 Introduction

Lignocellulose is a prominent biorefining raw material due to its wide availability and renewability, which helps to alleviate energy depletion (Somerville et al., 2010). In most lignocellulosic feedstocks, the proportions of cellulose, hemicellulose, and lignin are 30%–40%, 30%–35%, and 11%–25%, respectively. (Wang et al., 2016). Cellulose and hemicellulose separated from lignocellulose can be converted to high-value platform chemicals (e.g., 5-HMF and furfural) (Supplementary figure S1) (Bozell and Petersen, 2010).

Furfural is mainly obtained by hydrolysis and dehydration of xylan, which is abundant in hemicellulose and serves as a bridge between biomass and fuels, and chemicals. Furfural is widely used in oil refining, plastics manufacturing, pharmaceutical, and agrochemical industries, as well as a direct gasoline additive (Song et al., 2017; Luo et al., 2019; Song et al., 2020). Inorganic acids are utilized as the preferred catalysts for furfural production in the industry because furfural is produced in an acidic reaction system. High operating expenses, equipment corrosion, low furfural yields, and high energy consumption are all issues for this process (Mittal et al., 2017). Extraction of furfural from the reaction medium employing efficient catalysts or extraction solvents is a viable way for improving furfural yields. Some non-protonic solvents (e.g., tetrahydrofuran (THF), methyl isobutyl ketone (MIBK), and γ-pentyl lactone) are interesting solvents for furfural extraction because of their good ability to extract furfural. To extract furfural into the organic phase while minimizing side reactions in the aqueous phase, a biphasic reaction system is used. However, high reaction temperatures (150–230°C) are required for aqueous/organic biphasic systems (Romo et al., 2018). Ionic liquids with good solubility, chemical, and thermal stability, and enhanced catalytic activity were found to be alternative solvents, and polysaccharide dehydration in ionic liquids could be achieved at temperatures below 140°C (Wang et al., 2017; Wu et al., 2017). However, ionic liquids, are expensive and have poor biocompatibility.

Alternative deep eutectic solvents (DES) have been considered as a result of ongoing research on ionic liquids, which have similar properties to ionic liquids but are less expensive and entirely biodegradable (Hansen et al., 2021). DES are eutectic mixtures of hydrogen bond acceptor (HBA) and hydrogen bond donor (HBD) mixed in a certain ratio with a melting point lower than that of either component. For example, melting temperatures of chloroform and urea are 302°C and 135°C, respectively, and the resulting molar ratio of 1:2 chloroform/urea DES is liquid at 12°C (Abbott et al., 2003). DES has been used in biomass processing, for instance, fractionation and pretreatment of lignocellulose, as well as the catalytic conversion of carbohydrates (Satlewal et al., 2018).

The first half of this paper briefly covers DES and the mechanism of DES interacting with lignocellulose, with a focus on furfural synthesis. The second section summarizes the latest advances in the addition of various catalysts to DES systems for furfural production. This review also puts forward the challenges and perspectives for producing furfural from biomass using DES solvents.

## 2 Deep Eutectic Solvents

Deep Eutectic Solvents is a low melting point mixture of two or more component pairs consisting of HBD interacting with HBA (e.g., quaternary ammonium salts) via hydrogen bonding. Charge separation domains are promoted by hydrogen bonds (H-bonds) between HBA and HBD, showing a large decrease in the melting point of the DES mixture compared to the initial compounds, and the stronger H-bonds result in a bigger fall in melting point (Abbott et al., 2004). DES has been successfully applied in metallurgy and electrodeposition (Abbott et al., 2001; Abbott et al., 2006), biomass processing (Abbott et al., 2007; Francisco et al., 2012), fuel desulfurization (Jablonský et al., 2015), CO_2_ adsorption (Li et al., 2013), biodiesel purification (Li et al., 2008), biotransformation (Abbott et al., 2005), etc. since its first appearance in 2001 (Morrison et al., 2009). It can also be used as an effective extractant and has a wide range of applications in analytical sciences (Cunha and Fernandes, 2018), including chromatographic separation (Cai and Qiu, 2019), electrochemical analysis, liquid and solid sample decomposition (Chen Y.-L. et al., 2018), synthesis and modification of novel adsorbent materials (Hashemi et al., 2018).

### 2.1 Deep Eutectic Solvents Preparation and Principle

**FIGURE 1:**
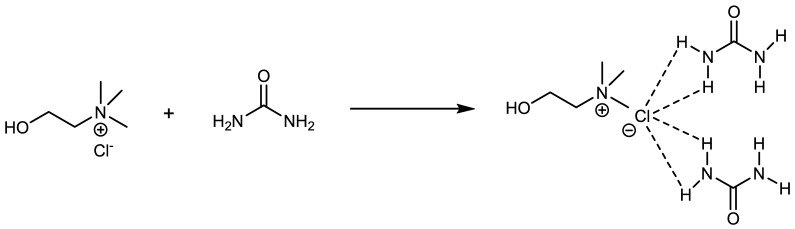
Hydrogen-bond interactions proposed for choline chloride/urea (1:2) eutectic mixture.

Autocorrelation intermolecular interactions, which are most likely induced by mixed entropy, van der Waals contacts, hydrogen bonding, and/or ionic bonding (Figure 1, exemplified for choline ChCl/urea 1:2), are responsible for the formation of DES (Abbott et al., 2003; Francisco et al., 2013; Hammond et al., 2016; Zahn et al., 2016; Mohd Zaid et al., 2017; Wagle et al., 2017; Delgado-Mellado et al., 2018; van Osch et al., 2019). Hydrogen bonds are the most important intramolecular bonds among these bonds between HBD and halide anions in DES (Perkins et al., 2014; Mbous et al., 2017). Hydrogen bonds are responsible for the generation of the room temperature liquid phase of DES (Smith et al., 2014), the enormous network of hydrogen bonds that generates the inherent qualities of DES, such as low melting point, low volatility, non-flammability, low vapor pressure, dipole nature, thermal stability, high solubility, and adjustability (Zhang et al., 2012; Florindo et al., 2014).

Heating and stirring, vacuum evaporation, grinding, and freeze-drying are the most common methods for preparing DES. The most common preparation method is heating and stirring, in which the components are mixed and stirred for a certain time at a set temperature (50°C–100°C) until a clear liquid is generated (Florindo et al., 2014; Yang et al., 2019). In addition, the excessive temperature may influence the formation of DES by this approach, resulting in the production of some contaminants (esters, HCl) (Florindo et al., 2014). Grinding is a better method when ChCl and carboxylic acid are used to obtain DES because the mixed components are ground in a mortar at room temperature until a homogeneous liquid is formed (Florindo et al., 2014). The evaporation method involves dissolving the components in water, evaporating most of the water under a vacuum at 50°C, and then drying it to a consistent weight in a desiccator (Dai et al., 2013). The components are dissolved in 5% water and then freeze-dried to generate a clear and transparent liquid (Gutierrez et al., 2009).

### 2.2 Deep Eutectic Solvents Categories

**FIGURE 2:**
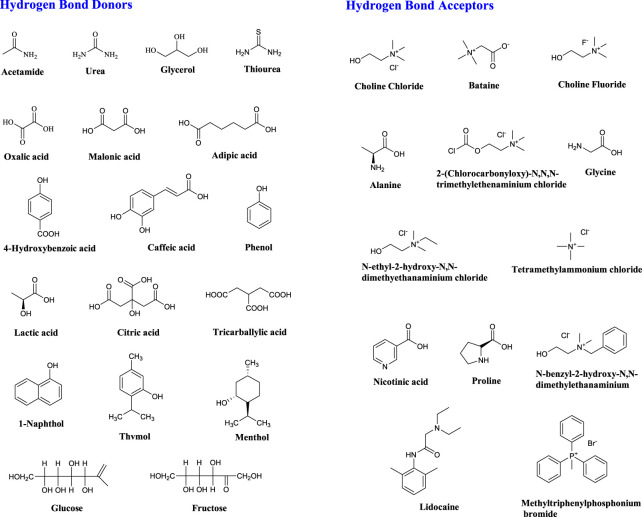
Typical structures of hydrogen bond donors (HBDs) and bond acceptors (HBAs) for DES synthesis.

Most DES are obtained from non-ionic substances, such as salts and molecular components (Figure 2) (Zhang et al., 2012). The general formula for DES is Cat^+^X^−^zY (Smith et al., 2014). DES was first proposed and classified into four categories by Abbott et al., in 2003 (Table 1) (Abbott et al., 2003) (Smith et al., 2014).

**TABLE 1:** The general formula for the classification of DES. Reprinted from ref. (Smith et al., 2014). Copyright 2014 American Chemical Society.

Entry	Type	Through-form	Composition	Terms
1	Type I	Cat^+^X^−^zMCl_x_	Organic and metal salts	M = Zn, Sn, Fe, Al, Ga, In
2	Type II	Cat^+^X^−^zMClx·yH_2_O	Organic salts and metal hydrates	M = Cr, Co, Cu, Ni, Fe
3	Type III	Cat^+^X^−^zRZ	Organic salts and HBD	Z = CONH_2_, COOH, OH
4	Type IV	MCl_x_^+^RZ = MCl_x-1_^+^RZ + MCl_x+1_^-^	Metal chloride hydrate and HBD	M = Al, Zn and Z = CONH_2_, OH

Cat^+^: Any ammonium (NR_4_^+^), phosphonium (PR_4_^+^), or sulfonium cation (SR_3_^+^) (Yiin et al., 2021).

X^−^: A Lewis base, generally a halide anion (F^−^, Cl^−^, Br^−^, I^−^, etc.).

z: The number of molecules Y that interact with the anion.

Type II DES employs hydrated metal halides, which are less expensive and less susceptible to atmospheric moisture than type I metal salts, making them popular in the industry (Smith et al., 2014). The most widely used DES is type III DES, which has the advantages of simple and low-cost preparation (Smith et al., 2014), being environmentally friendly and mostly biodegradable, not reacting with water, and having a high potential for lignocellulosic biomass processing, so this review will mainly focus on type III DES. In fact, in addition to the four types of DES mentioned above, type V has been developed, which is made up entirely of nonionic, molecular HBA, and HBD. These nonionic DES overcome the drawbacks of quaternary ammonium salt-based DES, such as expense, hydrophobicity, and viscosity, and exhibit the ability to recover and recycle DES by evaporation. However, the research aspect of V-type DES is not mature enough to be described here due to its late discovery (Abranches et al., 2019).

### 2.3 Catalytic and Solubilizing Effects of DES on Lignocellulose

#### 2.3.1 Dissolution

**FIGURE 3:**
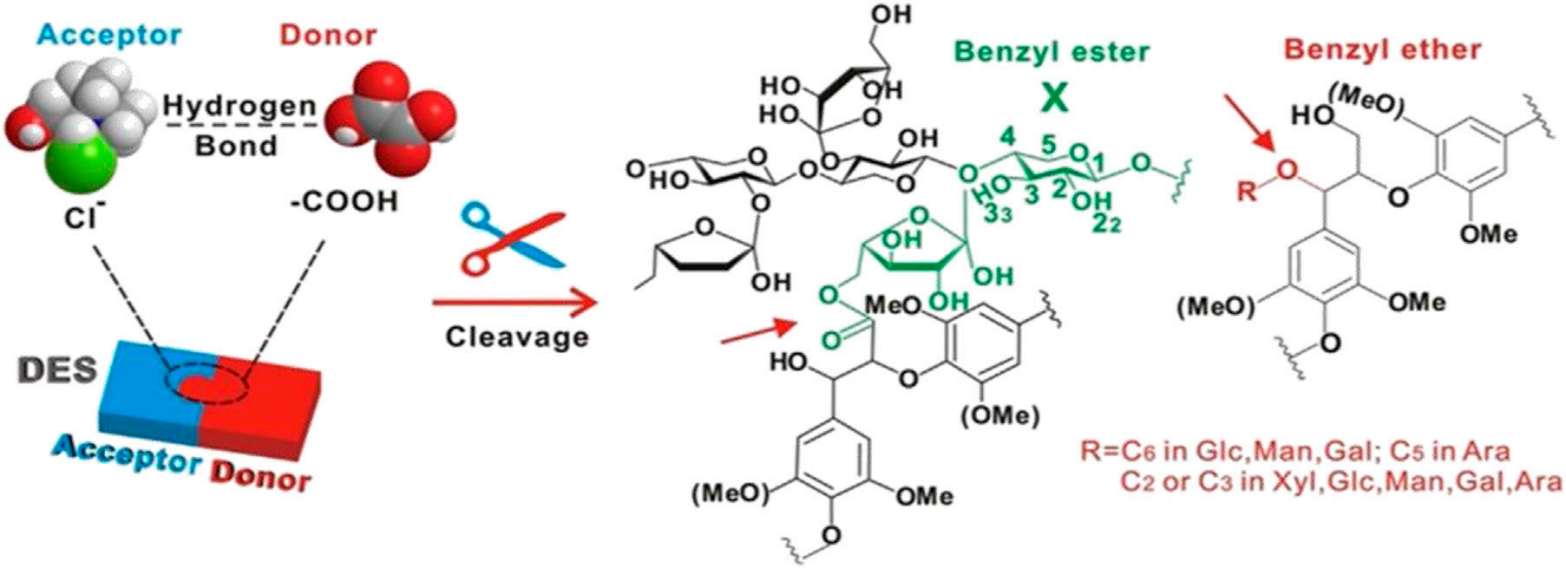
Disruption of lignin carbohydrate complexes with DES. Reprinted from ref. (Satlewal et al., 2018). Copyright 2018 Elsevier Inc.

Lignocellulosic biomass has a complicated structural and chemical mechanism for resistance to microbial and enzymatic deconstruction, as well as a natural resistance known as “biomass resistance,” which significantly inhibits biomass conversion into value-added products (Himmel Michael et al., 2007; Mettler et al., 2012). This “biomass resistance” is considered to arise mainly from lignin and lignin-carbohydrate complexes, which are cross-linked to carbohydrates (especially hemicellulose) through a network of strong covalent and hydrogen bonds with benzyl esters, benzyl ethers, and phenyl glycosides functional groups (Liu et al., 2017). The crosslinking degree of the lignin-carbohydrate complex is associated with increased cell wall stiffness and resistance to enzymatic digestion. To achieve an efficient deconstruction process, these crosslinks must be destroyed by the chemical hydrolysis of ester bonds (Grabber et al., 1998; McCann and Carpita, 2015). Strong hydrogen bonding ligands in ionic liquids (ILs) preferentially solubilize lignin and hemicellulose in biomass while maintaining biopolymer integrity (Carrozza et al., 2019). Inspired by the high solubilization capacity of ILs for lignocellulose, DES has attracted increasing interest as an inexpensive alternative to ILs. DES can deliver and accept protons, which enables the formation of hydrogen bonds with other compounds, thus enhancing their solubilization properties (Pandey et al., 2017). Acidic DES (HBD: lactic, malic, and oxalic acids) are very effective in lignin solubilization (Xu et al., 2016; Liu et al., 2017; Yiin et al., 2017; Liu et al., 2019; Ramesh et al., 2020; Yiin et al., 2021). One of the reasons why DES selectively dissolves lignin over cellulose is that both cellulose and DES have strong hydrogen bonding networks, solubilizing cellulose in DES requires dissociation and reorganization of both hydrogen-bonding networks to generate a thermodynamically more stable network. However, the cohesive energy of cellulose is so strong that it may prevent its solubilization in any DES (Vigier et al., 2015). As shown in Figure 3, chloride ions present in DES can establish hydrogen bonds with hydroxyl groups that are present in carbohydrates and lignin, disrupting the carbohydrate-lignin bonding connections and hydrolyzing the LCC to remove hemicellulose while also enhancing enzyme accessibility and hydrolysis yields (Satlewal et al., 2018; Lee and Wu, 2021). DES can effectively dissolve the lignin and remove amorphous cellulose from lignocellulose. As a result, DES can fractionate lignocellulose to promote hemicellulose and lignin utilization while also improving cellulose crystallinity.

#### 2.3.2 Catalysis

DES formed by combining ChCl and carboxylic acids is commonly used in the production of furan compounds. ChCl-based DES has been found to be effective for xylan solubilization in biomass pretreatment processes in recent studies (Chen et al., 2018b; Chen et al., 2018c; Shen et al., 2019). These DES based on ChCl-carboxylic acid can act as both solvents and catalysts. After lignin removal with the help of DES, hemicellulose, and cellulose were converted to furfural and hydroxymethylfurfural, respectively (Parsell et al., 2015). Hemicellulose is more readily depolymerized than cellulose (especially under acidic conditions) due to its non-crystalline nature (Brandt et al., 2013). After disruption of the hemicellulose polymer by chloride ions in DES, the carboxylic acids can provide the required protons to break down the sugar-1,4 glycosidic bonds in xylan to produce xylose, which can then be dehydrated to furfural (Enslow and Bell, 2012; Li et al., 2019). Unlike hemicellulose, the quantity and strength of hydrogen bonds established with cellulose determine cellulose’s dissolution (Lynam et al., 2017). To increase the output of furans, novel DES that can concurrently catalyze hemicellulose and cellulose must be discovered.

## 3 Catalytic Conversion of Biomass to Furfural in Deep Eutectic Solvents

The conversion of carbohydrates to furans is a hot research topic in the biorefining of lignocellulose (Li et al., 2016; Peleteiro et al., 2016; Fan et al., 2018). The acid-catalyzed hydrolysis and dehydration of pentosans in lignocellulose produces furfural. Two methods are generally used to produce furfural in the DES system. In the “two-step method” in which the lignin carbohydrate complex is destroyed by pretreatment, the hemicellulose-derived sugar is selectively dissolved from the biomass, and then the acidified aqueous DES solution is used to produce furfural from the xylose-rich pretreatment solution (Arora et al., 2021), and the “one-step method” in which the acidified DES is used to produce furfural by directly treating pentosan or biomass (Lee et al., 2019; Rusanen et al., 2021). The reaction can be further enhanced to promote furfural synthesis if Brønsted acid or Lewis acid catalyst is added to the DES system to some extent (Wang et al., 2018; Chen and Wan, 2019). Recent progress in the addition of various catalysts to DES systems for furfural production is shown in the chart.

### 3.1 Catalysis in Neat DES System

Furfural is produced by dehydration of xylose in an acidic medium (Enslow and Bell, 2012). HBD for various acidic complexing agents such as oxalic acid, citric acid, p-toluene sulfonic acid, trichloroacetic acid, and other acidic complexing agents with ionizable protons is named Brønsted acidic deep eutectic solvents (BADES) in acidic DES (ADES) (Qin et al., 2020). The acidic HBD can catalyze the hydrolysis of hemicellulose to form xylose or arabinose, which is further dehydrated to produce furfural by releasing three water molecules (Marcotullio and De Jong, 2010; Cai et al., 2014). The acidic strength of the acid catalyst/solvent is well known to play a critical role in the hydrolysis of hemicellulose (Choudhary et al., 2012; Wang et al., 2015; Delbecq et al., 2018), and it is thus critical to optimize and mediate ADES acidity (Kore and Srivastava, 2011). The higher the pH grew closer to 1, the larger the productivity of furfural, according to Arora et al. (2021). The yield of furfural was 85.4% after treatment of hemicellulose with ChCl: p-toluene sulfonic acid (p-TSA) = 1:1 and pH = 1 at 120°C for 1.5 h (Table 2, entry 1). When the pH was increased from 1 to 3, the yield of furfural reduced to 51.4% (Table 2, entry 2) (Arora et al., 2021). The effect of acidity on the yield of furfural was also confirmed by Cornelius et al. when comparing choline chloride-dicarboxylate-based low eutectic solvents. The increasing carbon chain length of dicarboxylic acid HBD boosts the electron feeding effect of alkyl groups, reducing the strength of the hydrogen bond formed between HBA and HBD as well as the hydrogen feeding capacity of DES, according to the reaction mechanism. Finally, because the concentration of H^+^ in the dehydration and hydrolysis reaction system is lowered, which is detrimental to furfural production, DES (oxalic acid) with short carbon chain dicarboxylic acid gives greater furfural yields (Lee et al., 2019). Lurii and others used ChCl/OA at 100°C to convert certain underappreciated terrestrial and marine biomass into furfural, yielding up to 72% (Table 2, entries 3–6), further demonstrating the superiority of oxalic acid as an HBD (Bodachivskyi et al., 2019). Although larger yields of furfural can be achieved without the use of a catalyst in a neat DES system, DES has drawbacks such as higher viscosity that cannot be overlooked. Although neat DES has absolute advantages for compounds with low water solubility, and better yields of furfural can be achieved in this system, practical issues such as time-consuming solvent transfer and inefficient mass transfer due to DES drawbacks such as higher viscosity cannot be neglected. Exploring some novel DES with low viscosity, high acidity, and good thermal stability is the main research topic in the future.

**TABLE 2:** Furfural production in a neat DES system (no catalyst).

Entry	Substrate	DES (mass ratio)	T (°C)	T(min)	Yield (%)	References
1	hemicellulose^a^	ChCl/p-TSA (1:1)	120	90	85.4	Arora et al. (2021)
2	hemicellulose^a^	ChCl/LA (1:1)	120	90	51.4	Arora et al. (2021)
3	corn husk	ChCl/OA (1:1)	100	120	52	Bodachivskyi et al. (2019)
4	Softwood	ChCl/OA (1:1)	100	300	55	Bodachivskyi et al. (2019)
5	*U. lactuca*	ChCl/OA (1:1)	100	120	60	Bodachivskyi et al. (2019)
6	*P. cruentum*	ChCl/OA (1:1)	100	240	72	Bodachivskyi et al. (2019)

LA, lactic acid; OA, oxalic acid.

a two-step method.

### 3.2 Catalyst in Deep Eutectic Solvents System

#### 3.2.1 Water

In biomass, water contributes to hemicellulose saccharification and xylose recovery. The addition of water in the DES system also reduces the viscosity of the DES (Dai et al., 2015), allowing for better DES penetration into the lignocellulosic matrix (New et al., 2019), improved mass transfer, and increased delignification, all of which aid hemicellulose extraction and subsequent furfural production (Loow et al., 2017). As a result, one of the most important influencing factors for hemicellulose conversion is the amount of water in the system. Annu et al. investigated the effect of different water contents on the yield of furfural in a deep eutectic solvent system. In the MIBK/DES biphasic system, higher furfural yields of 62% and 37.5% (Table 3, entry 1–2) were obtained for the sugar mixture and birch sawdust, respectively, when 32.9 wt% water was added to the microwave reactor (Rusanen et al., 2021). Water can promote furfural production, but too much water in the system can be harmful to getting enough furfural. When the amount of water injected exceeded 40 wt%, the yield of furfural decreased significantly (Rusanen et al., 2021). Excess water breaks the hydrogen bond between HBD and HBA and also promotes the formation of humins, thus obtaining a low furfural yield (Hammond et al., 2017; Gabriele et al., 2019). When comparing the yield of furfural before and after the addition of water, direct treatment of oil palm leaves with ChCl/OA aqueous solution (16.4 wt% H_2_O) at 100°C after the addition of 2 ml of water enhanced the production of furfural from 9.74% to 26.3%. (Table 3, entry 3) (Lee et al., 2019). A biphasic system was created using a ChCl: EG aqueous solution (30 percent water) combined with acetone (2:7), and xylose was reacted at 180°C for 30 min to get a high furfural yield of 75.6% (Table 3, entry 4) (Chen and Wan, 2019). The hydrogen bond between HBD and HBA can be weakened by the right amount of water employed in DES, making them more accessible and reactive. A deep eutectic solvent treatment with choline chloride-oxalic acid aqueous solution (16.4 wt% H_2_O) was added following ultrasonication of oil palm leaves to disrupt the LCC structure, yielding a furfural yield of 56.5% (Table 3, entry 5) (Lee et al., 2021). Unlike other similar studies, they used ultrasonic lignocellulose pretreatment. This technique produces acetic acid, which can efficiently increase furfural conversion while reducing reaction time (Lee et al., 2021). In the biphasic system, xylan was used as a starting material and 5 wt% water was added to ChCl/malic acid, then the system was heated by microwave at 150°C for 2.5 min to obtain a high yield of furfural (75%) (Table 3, entry 6) (Morais et al., 2020). This set of experiments was the shortest reaction time among these groups of similar experiments, and high yields of furfural were obtained. When compared to the traditional hydrothermal approach, either ultrasonic pretreatment or microwave heating can employ a shorter reaction time to get higher yields but raises the cost of industrial applications. The appropriate amount of water facilitates the production of furfural, but when the amount of water exceeds a certain threshold, the internal structure of DES is destroyed, and the interaction between DES and biomass is hampered, resulting in a reduction in furfural yield. Water mitigated several intrinsic flaws of DES and enhanced furfural yield, but this was limited. Compared to the reaction systems with the addition of Lewis acid, the furfural yield is lower when only DES acted as Brønsted acid without the presence of Lewis acid in the reaction because Lewis acid may catalyze the isomerization of xylose to xylulose, which is then further dehydrated to generate furfural. Finding an appropriate Lewis acid catalyst as well as deep eutectic solvents that are water-resistant is a pressing issue that must be addressed.

**TABLE 3:** Adding water in the DES system for furfural production.

Entry	Substrate	DES (mass ratio)	T (°C)	T(min)	Yield (%)	References
1	Xylose	ChCl/Gly (1:3)	160	10	62	Rusanen et al. (2021)
2	birch sawdust	ChCl/Gly (1:3)	170	10	37.5	Rusanen et al. (2021)
3	oil palm fronds	ChCl/OA (1:1)	100	135	26.3	Lee et al. (2019)
4	xylose^a^	ChCl/EG (1:2)	180	30	75.6	Chen and Wan, (2019)
5	oil palm fronds^a^	ChCl/OA (1:1)	120	60	56.5	Lee et al. (2021)
6	Xylan	ChCl/MA (1:3)	150	2.5	75	Morais et al. (2020)

Gly, glycerol; OA, oxalic acid; EG, ethylene glycol; MA, malic acid.

a two-step method.

#### 3.2.2 Metal Chlorides

Metal chlorides have been widely used as catalysts in the synthesis of furfural from lignocellulose (Chen et al., 2018b; Chen and Wan, 2019; Yu et al., 2019b). Metal chloride increases furfural yield due to the action of metal cations and Cl^−^ (Gravitis et al., 2001; Marcotullio and De Jong, 2010). For example, using ChCl/citric acid aqueous solution as the solvent and adding AlCl_3_·6H_2_O catalyst, 73.1% furfural yield (Table 4, entry 1) can be obtained from xylose and 68.6% (Table 4, entry 2) from xylan (Zhang and Yu, 2013). On aldose or aldose polymers, metal chlorides have an excellent isomerization impact. The addition of metal chloride to DES produced Lewis and Brønsted acid reaction medium. The metal chloride promoted the enolization of xylose and improved the selectivity and yield of furfural (Marcotullio and de Jong, 2011), and it catalyzes the dehydration of xylose to form furfural, through xylose isomerization to xylulose (Binder et al., 2010; vom Stein et al., 2011; Yang et al., 2012). When the biphasic system is applied in combination with the catalyst, excellent yields of furfural can be obtained. A high furfural yield of 70.3% (Table 4, entry 3) could be obtained by adding a specified amount of metal chloride as a catalyst to the DES (ChCl/OA)/MIBK biphasic system to react with eucalyptus (Wang et al., 2018). Zhang et al. studied the conversion of xylose to furfural by trivalent metal chlorides in ChCl-oxalic acid at 100°C, with the greatest furfural yields of 60.4% and 55.5% (Table 4, entries 4, 6) from xylose and xylan, respectively. AlCl_3_·6H_2_O was demonstrated to be the most efficient in generating furfural from xylose (Zhang et al., 2014). However, the thermal stability of ChCl/oxalic acid is poor at temperatures above 379 K (Zhang et al., 2014). A comparative investigation by Qiang et al. found that adding SnCl_4_·5H_2_O (Table 4, entries 7–10) to the ChCl/formic (Fa) system was the most successful (Yu et al., 2019a). Because the metal cation catalyzed the conversion of carbohydrates to furfural is related to its ionization potential, the furfural yields produced with different metal chlorides can vary. Although metal chlorides have good results for furfural yield, certain drawbacks should not be ignored, such as difficulty in separation and recovery, instability, and toxicity. Alkali metal salt catalysts have a limited function in furfural production, and combining alkali metal salts with acid catalysts in deep eutectic solvents improves furfural production. Metal chlorides dissolved in DES are difficult to be recovered for reuse, which is contrary to the principle of green chemistry. In industrial applications, new extractants are needed to separate metal chlorides from DES, or catalysts that are insoluble in DES are required.

**TABLE 4:** Adding metal chlorides in the DES system for furfural production.

Entry	Substrate	DES (mass ratio)	Catalyst	T (°C)	T(min)	Yield (%)	References
1	Xylose	ChCl/CA (2:1)	AlCl_3_·6H_2_O	140	25	73.1	Zhang and Yu, (2013)
2	xylan	ChCl/CA (2:1)	AlCl_3_·6H_2_O	140	35	68.6	Zhang and Yu, (2013)
3	eucalyptus	ChCl/OA (1:1)	AlCl_3_	140	90	70.3	Wang et al. (2018)
4	xylose	ChCl/OA (1:1)	AlCl_3_·6H_2_O	100	60	60.4	Zhang et al. (2014)
5	xylan	ChCl/OA (1:1)	FeCl_3_·6H_2_O	100	70	38.4	Zhang et al. (2014)
6	xylan	ChCl/OA (1:1)	AlCl_3_·6H_2_O	100	70	55.5	Zhang et al. (2014)
7	xylose	ChCl/FA (1:6)	SnCl_4_·5H_2_O	120	120	60.6	Yu et al. (2019a)
8	xylose	ChCl/FA (1:6)	AlCl_3_	120	30	39.8	Yu et al. (2019a)
9	xylose	ChCl/FA (1:6)	CeCl_3_·7H_2_O	120	30	39.1	Yu et al. (2019a)
10	xylose	ChCl/FA (1:6)	ZrCl_4_	120	30	32.8	Yu et al. (2019a)

FA, formic acid; OA, oxalic acid; CA, citric acid.

#### 3.2.3 Alkali Metal Halides

Alkali metal halides have been shown to increase the yield of furfural when added to solvent systems (water or non-polar solvents) (Enslow and Bell, 2015; Mellmer et al., 2019), mainly in biphasic systems to afford the “salting out” effect and thus increase the strong extraction of furfural from the aqueous phase into the organic phase. Similarly, they play a crucial role in the kinetics of xylose dehydration in acidic media. Eduarda and coworkers compared the differences of eight alkali metal salts added to DES to increase furfural yield: sodium chloride (NaCl), sodium bromide (NaBr), sodium iodide (NaI), potassium chloride (KCl), potassium bromide (KBr), potassium iodide (KI), lithium chloride (LiCl) and lithium bromide (LiBr). In comparison to the unadded halide salts, NaBr, NaI, KI, and LiBr exhibited improvement in furfural yield to a certain extent (Morais et al., 2021). The carbon cation is generated after the loss of water from xylose in the dehydration process of xylose to furfural, which is stabilized by the presence of halides. When a metal cation with significant hydrophilic characteristics interacts with the negatively charged xylose, it prevents the carbon cation from rehydration, stabilizing the generated carbon cation (Enslow and Bell, 2015). The carbon cation is a reactive intermediate that can undergo rearrangement to form 2,5-no-hydroxy sugars, which are then further dehydrated to produce furfural (Enslow and Bell, 2015). The halide anion serves as a stabilizing agent interacting with critical intermediate in the DES system, whereas the cation largely interacts with the chloride anion, and these interactions then influence xylose solvation and dehydration. The yield of furfural was then further optimized, and it was found that adding LiBr to ChCl/MA (5 wt% H_2_O) at 175.3°C for 1.74 min resulted in a high furfural yield of 89.5% (Table 4, entry 1) (Morais et al., 2021). Alkali metal halides can boost the yield of furfural by increasing the salting effect of the biphasic system, but in the complex lignocellulose structure, alkali metal salts cannot cooperate with DES to accelerate the isomerization of xylose and thus decrease the yield of furfural more efficiently. Alkali metal salt catalysts have a limited function in furfural production, and combining alkali metal salts with acid catalysts in DES improves furfural production.

#### 3.2.4 Solid Acid Catalysts

**FIGURE 4:**
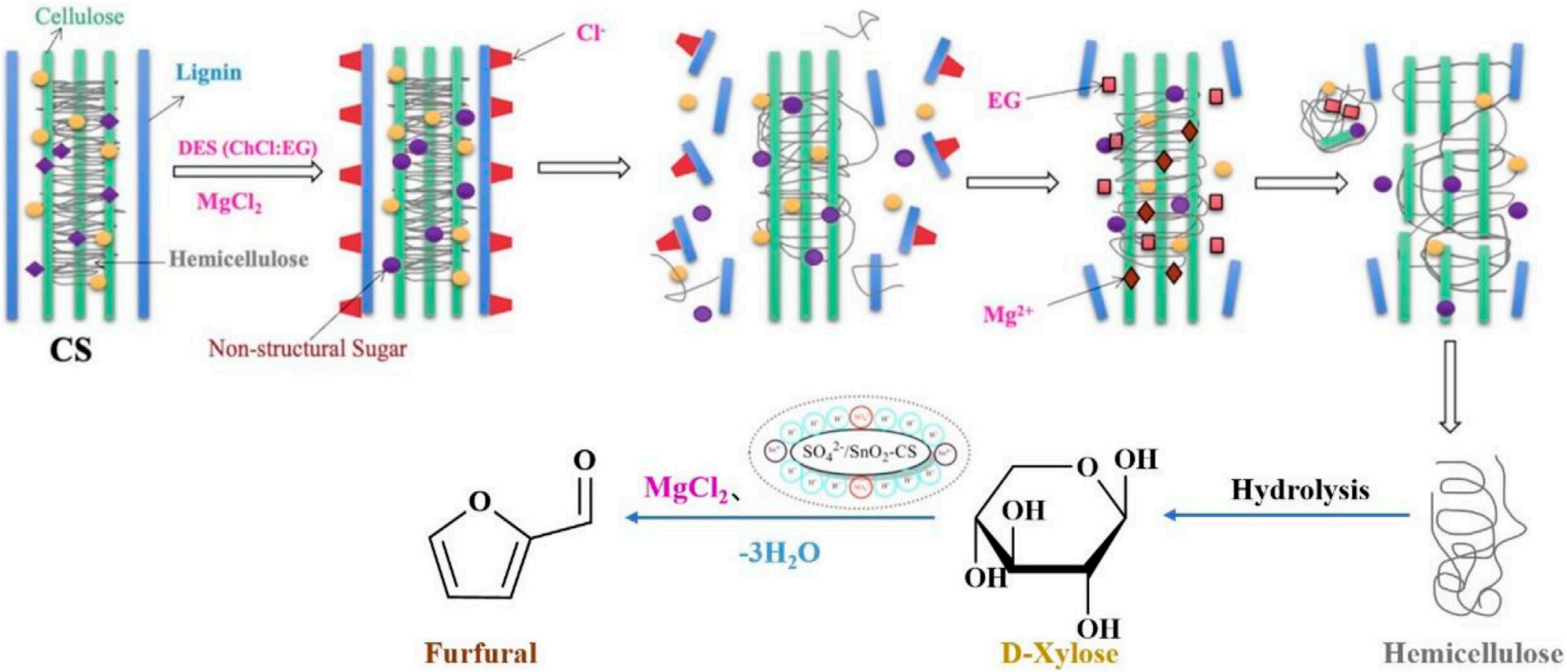
The catalytic mechanism for catalyzing corn stover into furfural and byproducts at 170°C for 0.5 h in ChCl: EG–water (20:80, v: v). Reprinted from ref. (Ji et al., 2021). Copyright 2021 Elsevier Ltd.

To avoid the potential corrosiveness and high energy cost of homogeneous catalysis (Li Y.-Y. et al., 2021), many solid acids have been used in furfural production which provides high furfural yields. A new heterogeneous catalyst B: LA-SG (SiO_2_) was prepared by immobilizing the deep eutectic solvent betaine/lactate on silica using the original tetraethyl silicate as the silica source by the sol-gel method. High furfural yields (45.3%) were obtained from corn cobs using the B: LA-SG (SiO_2_) catalyst (2.5 wt%) in water at 170°C for 0.5 h (Table 5, entry 2) (Li et al., 2022). However, the catalyst did not have an excellent catalytic performance in the production of furfural, owing to a lack of acidic sites in the catalyst. Lewis and Brønsted acid sites can isomerize aldose-ketose and accelerate furfural formation via ring dehydration (Li et al., 2022). Li et al. creatively used spent shrimp shells as a starting material to prepare sulfonated tin-based solid acids (Sn-SS) for the conversion of sugarcane bagasse (SB) to furfural in DES (ChCl/EG)-water at 170°C for 20 min, yielding 62.3% furfural (Table 5, entry 4) (Li et al., 2021a). The hydrogen bonds in SB were broken by ChCl/EG, and the Sn-SS solid acid could pretreat and depolymerize SB. Sn-SS contains Lewis and Brønsted acid sites, which can catalyze biomass into furfural. Unlike the previous study, Ji et al. directly used maize stover (CS) as a support to prepare the SO_4_^2-^/SnO_2_-CS catalysts for the production of furfural in ChCl: EG-water (20:80, v: v) at 170°C for 0.5 h, and a high furfural yield of up to 61.8% (Table 5, entry 5) was obtained (Ji et al., 2021). Compared with some traditional metal-based solid acids, biomass-based solid acids have the advantages of being less expensive, unique in structure, environmentally friendly, and easy preparation. The spongy and squishy shape of CS allows it to immobilize additional functional groups on its surface or inside it (Jiang et al., 2020). During the synthesis of furfural, chlorides can stabilize the transition state and intermediate structures, and reduce the occurrence of undesired side reactions (Figure 4) (Campos Molina et al., 2012; Ji et al., 2021). For instance, MgCl_2_ was added to the reaction system to improve the furfural yield up to 68.2% (Table 5, entry 6) (Ji et al., 2021). Both research groups use waste biomass as a source of catalysts, which is in keeping with the concept of sustainable and green chemistry. Solid catalysts, on the other hand, have difficulty recovering due to the higher viscosity of DES or insoluble by-products such as humins compounds clinging to them. Solid acid catalysts can flexibly tune the number of acidic sites to efficiently boost furfural yield, but the fabrication procedure is complicated and the catalyst stability is not very good, posing an economic barrier for practical application on a large scale. Reactions incorporating solid catalysts often require high energy, and most DES are thermally unstable and do not function optimally at high temperatures. Future research will focus on the development of novel highly efficient solid acid catalysts that are both inexpensive, robust, and facile to prepare, as well as deep eutectic solvents with improved thermal stability.

**TABLE 5:** Effect of the catalysts on the furfural yield in the DES system.

Entry	Substrate	DES (mass ratio)	Catalyst	T (°C)	T(min)	Yield (%)	References
1	xylan	ChCl/MA (1:3)	LiBr	157.3	1.74	89.5	Morais et al. (2021)
2	corncob	Betaine/LA	DES-SG (SiO_2_)^a^	170	30	45.3	Bodachivskyi et al. (2019)
3	corncob	Betaine/LA	SG(SiO_2_)^a^	170	30	38.7	Bodachivskyi et al. (2019)
4	sugarcane bagasse^b^	ChCl/EG (1:2)	Sn-SS^c^	170	20	62.3	Li et al. (2021a)
5	corn stover	ChCl/EG (1:2)	SO_4_^2-^/SnO_2_-CS^a^	170	30	61.8	Ji et al. (2021)
6	corn stover	ChCl/EG (1:2)	SO_4_^2-^/SnO_2_-CS, MgCl_2_	170	30	68.2	Ji et al. (2021)
7	xylose	ChCl/Gly (1:2)	HCOOH	180	30	58.3	Li et al. (2021b)
8	native cellulose	ChCl/OA (1.5:1)	TiO_2_	140	30	53.2^d^	da Silva Lacerda et al. (2015)

MA, malic acid; EG, ethylene glycol; LA, lactic acid; Gly, glycerol; OA, oxalic acid.

a SG: sol–gel method.

b Two-step method.

c SS: Shrimp shell.

d Yield of HMF + Furfural.

#### 3.2.5 Other Catalysts

Other additions, such as oxides, organic acids, and heterogeneous catalysts, can boost furfural yields in addition to the foregoing catalysts. The addition of HCOOH has been shown to give a high furfural yield (58.3%) from biomass-derived xylose in the ChCl: Gly-water system within 30 min at 180°C (Table 5, entry 7), compared to the reaction system without the addition of catalyst (Li et al., 2021b). The simplest organic acid, HCOOH, can be employed as a dehydration catalyst in the production of furfural, overcoming the main disadvantage of corrosive inorganic acid catalysts to some extent. The addition of oxides can also improve the furfural yield, Viviane et al. mixed ChCl/OA, sulfolane, substrate, TiO_2_, and water to catalyze lignocellulosic materials and obtained the greatest yield of 53.2% for HMF + furfural (Table 5, entry 8) (da Silva Lacerda et al., 2015).

## 4 Conclusion and Perspective

This review summarizes the most recent progress on the conversion of biomass to furfural using various catalysts in DES systems. Unlike previous aqueous and organic systems, which required high temperatures and had low furfural yields, the DES/biphasic systems (DES/organic solvent) can achieve high furfural yield at lower temperatures and avoid the formation of humins by subsequent side reactions due to high furfural concentration. The ability to create furans at higher substrate concentrations and superior lignocellulose separation are two advantages of deep eutectic solvents. The addition of an appropriate catalyst promotes the dehydration of xylose/xylan into furfural with higher yields. At present, the manufacture of high-yield furfural utilizing a deep eutectic solvent is still in its initial stages of development, with numerous challenges and innovation constraints.

(1) When employing an organic phase and a deep eutectic solvent for a biphasic system, large yields can only be achieved in the laboratory, but it is very difficult for industrial applications. The extraction efficiency of organic solvents is high, but recovery requires energy-intensive devices and most organic solvents are toxic.
(2) It is necessary and cumbersome to firstly transform hemicellulose of lignocellulose into xylan with high yields because lignocellulose has recalcitrant in order to obtain high furfural yield, which results in the loss of some hemicellulose.
(3) Recycling deep eutectic solvents is a more energy-efficient and environmentally benign solution from the standpoint of sustainable development. On the other hand, deep eutectic solvents are polluted and cannot be reused due to their high viscosity and the fact that a portion of the catalyst will stay in DES.
(4) Some deep eutectic solvents have poor thermal stability, while some solid catalysts (e.g., Nb_2_O_5_) can only function at higher temperatures, resulting in a suboptimal reaction.

Recently, it has been discovered that there exists hydrophobic DES, which can extract furfural from water efficiently and can replace the role of organic solvent in the DES/biphasic system, thus alleviating the high energy consumption due to organic solvent. It is urgent to develop novel deep eutectic solvents and to explore a sustainable and more cost-effective biorefinery method for producing furfural from lignocellulosic biomass.
