# Supplementary figures and images for: Recent Advances in the Catalytic Conversion of Biomass to Furfural in Deep Eutectic Solvents
Source: Front Chem. 2022 May 9;10:911674. doi: 10.3389/fchem.2022.911674 (PMC9124943; doi:10.3389/fchem.2022.911674)

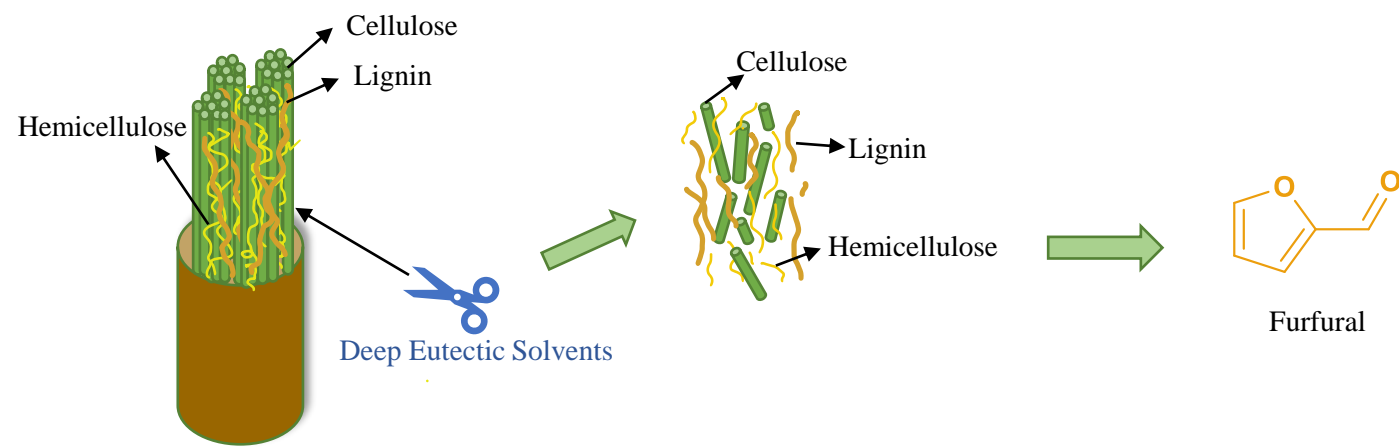

Supplement: Supplementary file 1 [file DataSheet1.PDF]
